# ING1b negatively regulates HIF1*α* protein levels in adipose-derived stromal cells by a SUMOylation-dependent mechanism

**DOI:** 10.1038/cddis.2014.577

**Published:** 2015-01-22

**Authors:** N Bigot, C Guérillon, S Loisel, N Bertheuil, L Sensebé, K Tarte, R Pedeux

**Affiliations:** 1 INSERM U917, Microenvironnement et Cancer, Rennes, France; 2 Université de Rennes 1, Rennes, France; 3 Etablissement Français du Sang Bretagne, Rennes, France; 4 Service ITeCH, CHU Pontchaillou, Rennes, France; 5 Etablissement Français du Sang Pyrénées Méditerranée; 6 Université Paul Sabatier, Toulouse, France; 7 UMR5273-INSERM U1031, Toulouse, France

## Abstract

Hypoxic niches help maintain mesenchymal stromal cell properties, and their amplification under hypoxia sustains their immature state. However, how MSCs maintain their genomic integrity in this context remains elusive, since hypoxia may prevent proper DNA repair by downregulating expression of BRCA1 and RAD51. Here, we find that the ING1b tumor suppressor accumulates in adipose-derived stromal cells (ADSCs) upon genotoxic stress, owing to SUMOylation on K193 that is mediated by the E3 small ubiquitin-like modifier (SUMO) ligase protein inhibitor of activated STAT protein *γ* (PIAS4). We demonstrate that ING1b finely regulates the hypoxic response by triggering HIF1*α* proteasomal degradation. On the contrary, when mutated on its SUMOylation site, ING1b failed to efficiently decrease HIF1*α* levels. Consistently, we observed that the adipocyte differentiation, generally described to be downregulated by hypoxia, was highly dependent on ING1b expression, during the early days of this process. Accordingly, contrary to what was observed with HIF1*α*, the absence of ING1b impeded the adipogenic induction under hypoxic conditions. These data indicate that ING1b contributes to adipogenic induction in adipose-derived stromal cells, and thus hinders the phenotype maintenance of ADSCs.

Human mesenchymal stem/stromal cells (MSCs) are able to self-renew and differentiate into various cell types. Recently, MSCs have been developed as tools for tissue engineering and cell-based therapies^1^ in particular owing to their trophic and immunosuppressive activities.^2^ Conventionally, the bone marrow MSCs (BM-MSCs) and the adipose-derived stem/stromal cells (ADSCs) have constituted the main sources of MSCs for clinical use. These cells are expanded *in vitro* prior to their application; however, this long-term culture may allow the emergence of senescence and phenotypic alterations, rendering MSCs unsuitable for clinical purposes.^3^

To overcome these issues, MSC culture in conditions mimicking hypoxic niches has been tested.^4^ Low O_2_ tensions promote MSC growth, survival and maintain their self-renewing multipotent state.^5^ However, how hypoxia (1% O_2_) affects MSC behavior is unclear. Responses to hypoxia are mainly mediated by hypoxia inducible factors (HIFs). HIF1, 2 and 3*α* subunits, are constitutively degraded in normoxia and stabilized in hypoxia. Consequently, when stabilized they can dimerize with HIF1*β*, and then translocate into the nucleus to modulate the expression of selected genes. HIF1*α* is highly expressed in MSCs, controls their metabolic fate and maintains them in an undifferentiated state.^6^ HIF1*α* has also been shown to delay the occurrence of senescence in MSCs, by repressing E2A and p21 expression.^7^

The inhibitors of growth (ING) family genes act as readers of the epigenetic histone code. Among them, ING1 has been described as a type II tumor suppressor, regulating cell growth, DNA repair, apoptosis, chromatin remodeling and senescence.^8^ To some extent, ING1 and HIF might have opposite effects, (e.g. on tumor progression). Indeed, HIF1*α*, unlike ING1 that inhibits angiogenesis, promotes angiogenesis.^9^ Furthermore, p53, a well-known ING1b interactor, and HIF1*α* have been shown in several studies to have antagonistic effects. Following DNA damage, p53 induces apoptosis and inhibits survival of cells by reducing activity and levels of HIF1*α*.^10, 11^

So far, ING4 has been shown as the only ING protein to regulate the hypoxic response. Indeed, by interacting with HIF prolyl hydroxylase 2 (HPH-2), ING4 has been described to repress some HIF1*α* activities under hypoxic conditions.^12^ Here, we show that ING1b accumulates in ADSCs following DNA damage in hypoxia. According to the opposing roles of ING1b and HIF1*α*, we hypothesized that ING1b could interfere with HIF1*α* and participate in the conservation of the genomic integrity of MSCs. Mechanistically, we found that ING1b interacted with HIF1*α* and promoted its proteasomal degradation in hypoxia. SUMOylation of ING1b played a role since the unSUMOylated form of ING1b was unable to trigger HIF1*α* degradation. The E3 small ubiquitin-like modifier (SUMO) ligase protein inhibitor of activated STAT protein *γ* (PIAS4) participated in HIF1*α* degradation and ING1b accumulation following a genotoxic stress in 1% O_2_. ING1b, subsequently, took part in decreasing PIAS4 levels after DNA damage. Finally, we report that ING1b by decreasing HIF1*α* level modulated ADSC differentiation potential. These data indicate that ING1b, according to its SUMOylation status, regulates the hypoxic response by contributing to the HIF1*α* degradation, and therefore may impede HIF1*α*-related effects on the maintenance of ADSCs stem cell character.

## Results

### ING1b protein levels increase following genotoxic stress in ADSCs cultured under hypoxic conditions

**Figure 1:**
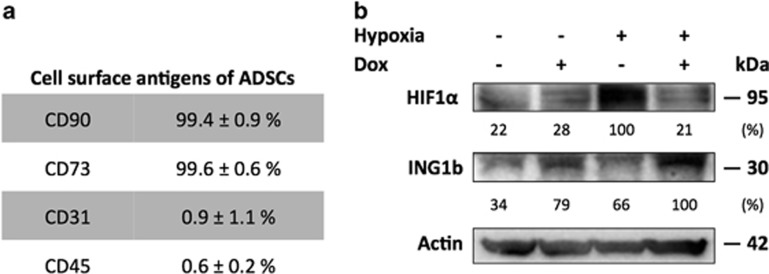
Adipose derived stromal cells (ADSCs) express higher levels of ING1b in hypoxia following genotoxic stress. (**a**) Characterization of ADSCs at P1. Expression level is expressed as a percentage of positive cells targeted for CD90, CD73, CD31 and CD45 compared with their isotypic controls (mean±S.D., *n*=3). (**b**) ING1b increases while HIF1*α* decreases in ADSCs under hypoxia. ADSCs were incubated for 24 h in normoxia (21% O_2_) or hypoxia (1% O_2_) (indicated by a ‘–' and a ‘+', respectively), prior to a doxorubicin treatment (Dox) at a final concentration of 10 *μ*M for 3 h. Cells were maintained in normoxia or hypoxia, and harvested 24 h later. Whole-cell extracts were fractionated by SDS-PAGE and probed with the indicated antibodies. The Western blot shown is a representative figure of three experiments

**Figure 2:**
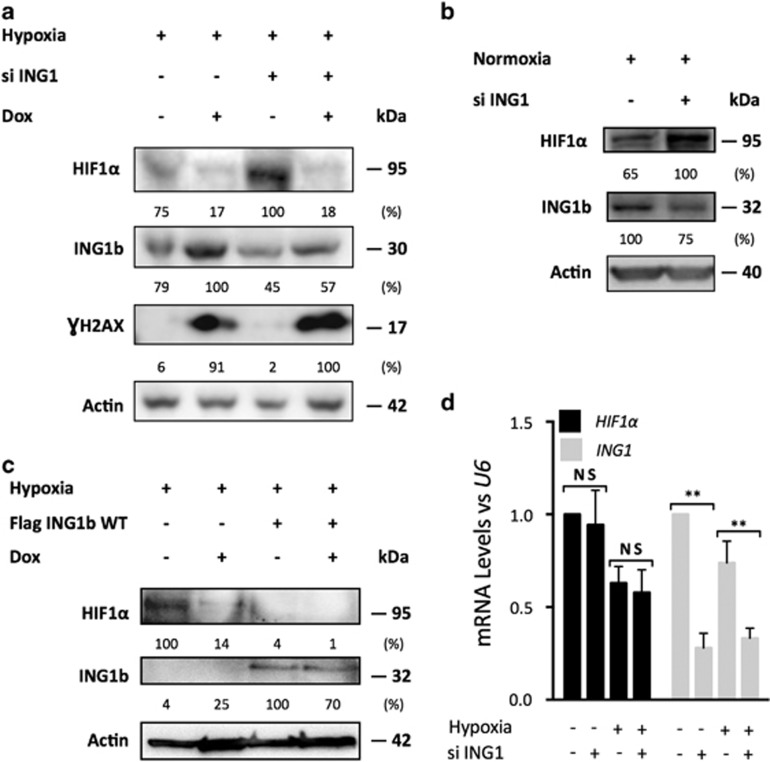
ING1b in hypoxia induces HIF1*α* protein degradation following genotoxic stress. (**a**) HIF1*α* increases when ING1b is depleted. ADSCs were incubated for 24 h in hypoxia prior to ING1b knockdown. Forty-eight hours later cells were either incubated or not with Dox for 3 h. Twenty-four hours later, cells were harvested and proteins analyzed as described in **b**. γH2AX marks DNA damage induced by doxorubicin. (**b**) ING1b knockdown in normoxia increases HIF1*α* levels. ADSCs were submitted to an ING1b targeting siRNA under 1% O_2_ for 72 h. Samples were used as in **a.** (**c**) HIF1*α* protein levels are reduced when ING1b WT is overexpressed in ADSCs in hypoxia. Following 24 h in hypoxia, ADSCs have been transiently transfected with either an empty pcDNA 3.1 vector or with the pcDNA 3.1 Flag ING1b WT plasmid in hypoxia. Twenty-four hours later, cells were incubated for 3 h with Dox. Twenty-four hours later, whole-cell lysates were analyzed using Western blot. (**d**) HIF1*α* transcription is not modulated by ING1b in ADSCs. ADSCs incubated or not in hypoxia were submitted to an ING1b silencing for 48 h. *HIF1α* and *ING1b* expressions were analyzed by RT Q-PCR. Each sample was normalized to *U6* level. Bars represent mean±S.E.M., *n*=3, non significant (NS) *P*<0.01 (**) in an unpaired Student's *t*-test. The Western blots shown are representative of three experiments

At first, we aimed at evaluating the behavior of MSCs in response to DNA damage, in normoxia and hypoxia. For that purpose, we used fully characterized ADSCs isolated from human lipoaspirates (Supplementary Figure 1). Over 98% of cells were positive for CD90 and CD73 and <2% were positive for CD31 and CD45^13^ (Figure 1a). To investigate the associated effects of variable O_2_ tensions and genotoxic stress on ADSCs, we placed them either under 21% O_2_ or under 1% O_2_, in the presence of doxorubicin (Figure 1b). As expected, HIF1*α* accumulated in hypoxic ADSCs but its expression decreased after the induction of DNA damage, while we observed an increase of ING1b. Following ING1 silencing, ADSCs displayed greater HIF1*α* protein levels in hypoxia, arguing for a specific relationship between HIF1*α* and ING1b (Figure 2a). Interestingly, ING1b knockdown led to HIF1*α* increase in normoxia as well (Figure 2b). These effects did not appear to be dependent on ING4, since ING1 silencing had no noticeable effect on ING4 expression (Supplementary Figure 2). The ING1b depletion did not affect HIF1*α* mRNA levels 48 h after siRNA transfection, suggesting that HIF1*α* regulation by ING1b may occur later at the mRNA level or rather at the protein level (Figure 2d). This inverse relationship between ING1b and HIF1*α* was confirmed using transient overexpression of ING1b in ADSCs under hypoxic conditions, leading to a decrease of HIF1*α* (Figure 2c). Together, these findings suggest that under hypoxic conditions, in response to a genotoxic stress, ING1b may trigger HIF1*α* degradation in ADSCs.

### SUMOylation of ING1b triggers HIF1*α* degradation

**Figure 3:**
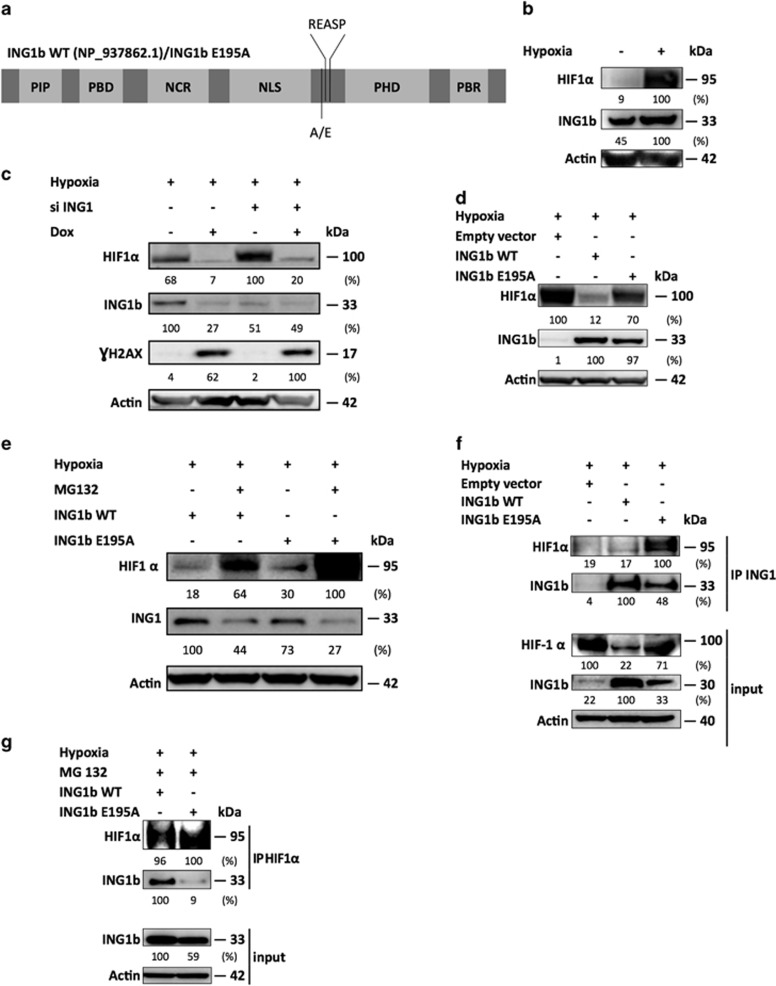
ING1b and HIF1*α* interact with each other in hypoxia, but SUMOylated ING1b favors HIF1*α* degradation. (**a**) Protein structure of Human ING1b. Human ING1b WT and non SUMOyable ING1b E195A proteins domains: NCR, novel conserved region; NLS, nuclear localization signal; PBD, partial bromo domain; PBR, polybasic region; PHD, plant homeodomain; PIP, PCNA-interacting protein. (**b**) Untransfected U2OS cells express HIF1*α* in hypoxia. Whole-cell extracts from untransfected U2OS cells cultured for 24 h in 1% O_2_ atmosphere were fractionated by SDS-PAGE and probed with the indicated antibodies. (**c**) ING1b induces HIF1*α* decrease in U2OS cells. Twenty-four hours after they were incubated in hypoxia, wild-type U2OS cells were transfected with siRNAs targeting ING1b as described in Figure 1c. (**d**) HIF1*α* decreases in ING1b WT overexpressing U2OS cells, while ING1b E195A rescues HIF1*α* protein levels. Stably transfected U2OS cells with pcDNA 3.1 (empty vector), pcDNA 3.1 ING1b WT or pcDNA 3.1 ING1b E195A were cultured for 24 h in 1% O_2_. Cells were harvested, and whole-cell lysates were analyzed in Western blot. (**e**) An inhibition of the proteasomal activity in hypoxia leads to a HIF1*α* increase and an ING1 decrease. Stably transfected ING1b WT and ING1b E195A U2OS cells were cultured for 24 h in hypoxia and then incubated with the proteasome inhibitor MG132 (20 *μ*M) for 24 h in hypoxia. Whole-cell lysates were used in Western blot experiments. (**f** and **g**) ING1b interacts with HIF1*α* in hypoxia. After 24 h in hypoxia, U2OS cells stably transfected with ING1b WT, ING1b E195A or empty vector were collected for ING1b immunoprecipitations (**f**). To maintain sufficient HIF1*α* protein levels, because of ING1b-related pro-degrading effects, U2OS cells were incubated again for 24 h with MG132 (20 *μ*M) in hypoxia and HIF1*α* immunoprecipitations were performed (**g**). All the Western blots shown are representative of three experiments

We hypothesized that the ING1b effect on HIF1*α* may be associated with post-translational modifications. Indeed, the phosphorylation of ING1b is needed for its activity and stabilization.^14^ Recently, ING1 was seen to be SUMOylated on K193,^15^ and we demonstrated that ING1b may be mono or diSUMOylated in ADSCs as well (Supplementary Figure 3). On the other hand, data on HIF1*α* SUMOylation are scarce, and the consequences of this modification remain unclear.^16, 17^ Accordingly, we thought that ING1b SUMOylation could be required for its HIF1*α* pro-degrading effects. A SUMOylation site prediction tool (SUMOplot Analysis Program http://www.abgent.com/sumoplot) identified the AKAE motif (192–195), located at the junction of the last nucleolar targeting sequence of the NLS domain and the REASP amino acid motif. As a consequence, a SUMOylation defective mutant was generated and named ING1b E195A (Figure 3a). In U2OS cells, ING1b expression is higher than in ADSCs (Supplementary Figure 4). As observed in ADSCs, HIF1*α* protein levels under hypoxic conditions increased in untransfected U2OS cells (Figure 3b) and a knockdown for ING1b led to an increase of HIF1*α* as well (Figure 3c). Moreover U2OS cells are easier to transfect than ADSCs. Therefore, U2OS were used to study the interactions of ING1b with HIF1*α*. Thus, U2OS cells were chosen to be stably transfected with pcDNA 3.1 ING1b WT or pcDNA 3.1 ING1b E195A constructs, to use a model that express moderate levels of ING1b WT or ING1b E195A proteins. Interestingly, in hypoxia, U2OS harboring ING1b WT exhibited low HIF1*α* levels, compared with ING1b E195A, which failed to efficiently trigger HIF1*α* degradation (Figure 3d). Furthermore, ING1b E195A strongly interacted with HIF1*α* under hypoxic conditions compared with ING1b WT (Figure 3f). Assuming this lack of detectable protein interactions between ING1b WT and HIF1*α* might be due to a rapid degradation by the proteasome (Figure 3e), HIF1*α* immunoprecipitation was performed in the presence of the MG132 proteasome inhibitor (Figure 3g). By impeding proteasomal activity, the interaction between the ING1b WT and HIF1*α* proteins was detected.

The immunoprecipitation experiments suggested that ING1b E195A, unlike ING1b WT, might maintain interactions with HIF1*α*. This indicates that as long as ING1b is not SUMOylated, interactions with HIF1*α* are conserved under hypoxic conditions and therefore may prevent degradation

### The PIAS4-dependent stabilization of ING1b promotes PIAS4 decrease in return

**Figure 4:**
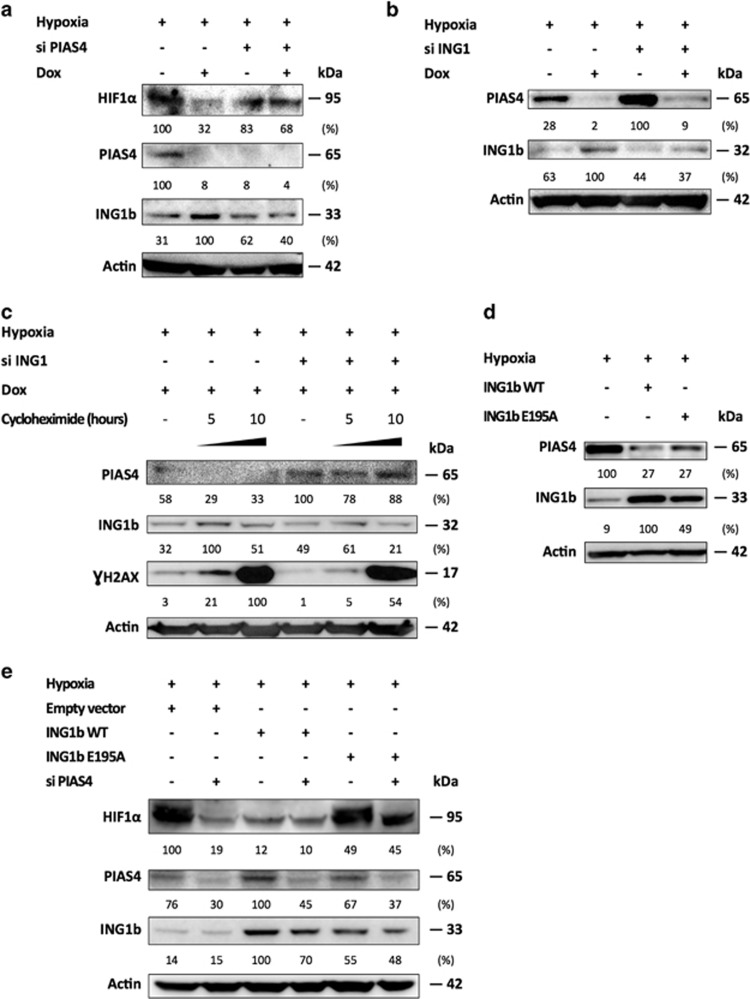
ING1b and HIF1*α* stabilizations depend on PIAS4, but ING1b inhibits PIAS4 in return. (**a**) PIAS4 depletion leads to reduced ING1b and HIF1*α* protein levels. Experiment was performed as in Figure 1c, except that PIAS4 was silenced. (**b**) ING1b knockdown increases PIAS4 protein levels in ADSCs cultured in hypoxia. Experiment was designed as described in Figure 1c. (**c**) Decrease of PIAS4 protein levels, following doxorubicin treatment, depends on ING1b in ADSCs cultured in hypoxia. ADSCs were incubated in 1% O_2_ for 24 h prior to ING1b knockdown. Twenty-four hours later, cells were submitted to doxorubicin treatment (Dox). Three hours later media were replaced with media containing cycloheximide (10 *μ*g/ml) or not for 0, 5 or 10 supplementary hours to inhibit translation. Whole-cell extracts were run in Western blot with the indicated antibodies. γH2AX indicates the occurrence of DNA damage. (**d**) Stable expression of ING1b WT leads to decreased PIAS4 protein levels in U2OS. Stably transfected U2OS cells expressing either ING1b WT or ING1b E195A were placed in hypoxia for 24 h. Whole-cell lysates were used in Western blot. (**e**) Defective ING1b SUMOylation mutant maintains HIF1*α* protein levels after a PIAS4 silencing. Stably transfected U2OS cells were cultured in hypoxia for 24 h prior to a PIAS4 knockdown. Cells were harvested 48 h later and samples were used in Western blot. All the Western blots shown are representative of three experiments

Since we have recently described that the E3 conjugating enzyme PIAS4 participated in the ING1b SUMOylation,^15^ we hypothesized that PIAS4 could be involved in the shift from a conservation to a degradation of HIF1*α* by regulating ING1b. We therefore investigated the role of PIAS4, a SUMO E3 ligase characterized to enhance ING1 SUMOylation and its activity,^15^ but also described to regulate HIF1*α*.^16, 18^ In Figures 4a and b, we showed that under hypoxic conditions, the induction of a genotoxic stress increased ING1b levels and led to a decrease of PIAS4 (Figure 4a, lane 2). Interestingly, a PIAS4 knockdown reduced ING1b protein levels in hypoxia after doxorubicin treatment, suggesting that ING1b accumulation in ADSCs relied on PIAS4 (Figure 4a, lane 4).^19^ By contrast, we observed that ADSCs submitted to an ING1b knockdown displayed increased PIAS4 levels in control cases and it was also the case following doxorubicin treatment but to a lesser extent (Figure 4b). Then, we aimed to determine whether the PIAS4 decrease, related to the genotoxic stress in hypoxia, could be dependent on ING1b. Thus, we managed to block the protein translation with cycloheximide and we evaluated if the doxorubicin treatment could modulate PIAS4 protein levels (Figure 4c). By inhibiting the protein translation, we noticed a PIAS4 protein decrease following the doxorubicin treatment, but also that PIAS4 protein levels were less impaired when ING1b was knocked down. This suggested that doxorubicin reduced PIAS4 at a protein level through ING1b activities and that increasing levels of ING1b might oppose to PIAS4 (Figure 4c). In a same manner, U2OS cells expressing ING1b WT, tended to feature lower amounts of PIAS4 compared with the ING1b E195A expressing cells in hypoxia (Figure 4d). Altogether, these data suggest that the increase of ING1b protein levels that occurs after this DNA damage may be attributable to a prior PIAS4-dependent SUMOylation. This then appears to allow the activated form of ING1b to contribute to the reduction of PIAS4 protein levels.

### PIAS4 silencing leads to reduced HIF1*α* protein levels

SUMOylation has been shown to be a post-translational modification altering the stability or activity of several factors in the hypoxic pathway, including HIF1*α*. SUMOylation has been described to either stabilize or enhance HIF1*α* signaling,^20^ its transcriptional activity^16, 18, 21^ or to participate in the proteasomal degradation of HIF1*α* in hypoxia.^17, 22^ To clarify the role of PIAS4, ADSCs and U2OS cells were knocked down for PIAS4 and treated with doxorubicin during hypoxia. Thereby, we observed in ADSCs that ING1b accumulation that is required for triggering HIF1*α* degradation, seems to be PIAS4 dependent following doxorubicin treatment. However, this PIAS4 silencing slightly reduced the HIF1*α* levels as well (Figure 4a, lane 3). Interestingly, the doxorubicin deleterious effects on HIF1*α* were reduced in a substantial way when PIAS4 was silenced compared with its control (Figure 4a, lanes 2 and 4). In hypoxia, a PIAS4 knockdown in U2OS cells expressing ING1b WT and ING1b E195A demonstrated that PIAS4 was required for the stability of HIF1*α* (Figure 4e, lanes 1 and 2). Of note, in cells expressing ING1b WT, depletion of PIAS4 had no effect on HIF1*α* levels, hinting again that HIF1*α* depends on ING1b for its degradation and PIAS4 for its stabilization (Figure 4e, lanes 3 and 4). Low HIF1*α* level (lane 4), could be the result of a balance between lower ING1b favoring HIF1*α*, and low PIAS4 hindering HIF1*α* accumulation. Still, ING1b E195A cells featured a less substantial HIF1*α* degradation when PIAS4 was lacking, further suggesting that unSUMOylated ING1b may maintain HIF1*α*, regardless of the HIF1*α* SUMOylation status. Taken together, these results suggest that HIF1*α* protein stability in hypoxia does not only rely on ING1b levels, but also on ING1b SUMOylation, according to PIAS4 silencing experiments. Moreover, PIAS4 appears to be needed for HIF1*α* protein conservation as well following doxorubicin treatment.

### ING1b and HIF1*α* modulate ADSCs fate

**Figure 5:**
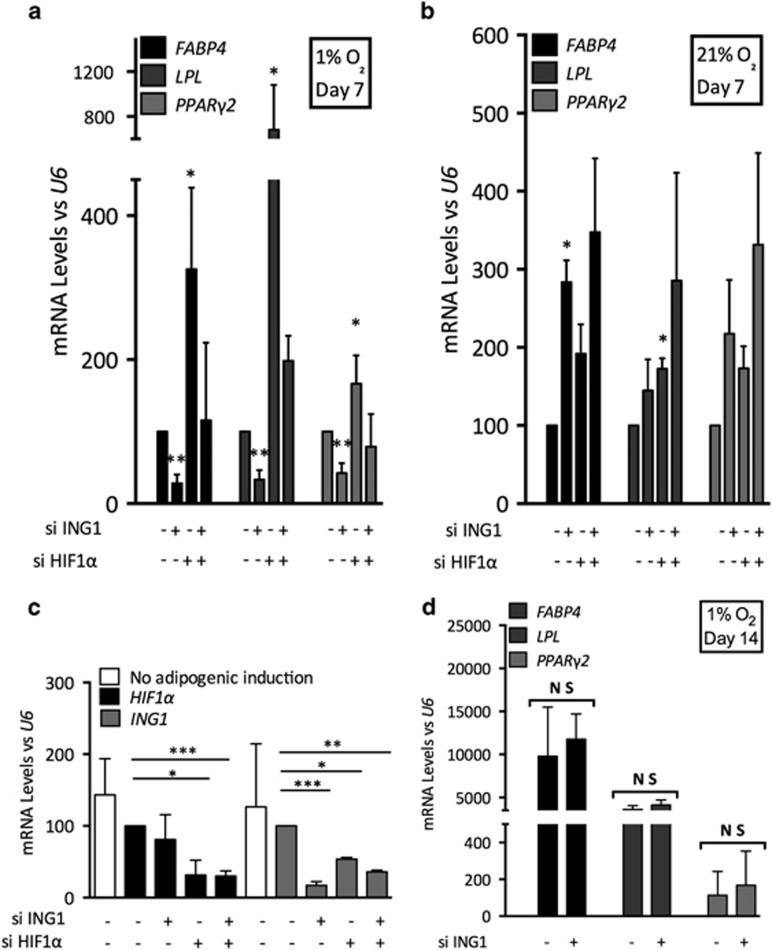
ING1b modulates adipogenesis process in hypoxia. ING1b and HIF1*α* have opposite effects on adipogenic differentiation of ADSCs until 7 days of induction. (**a**) ADSCs were cultured for 24 h in hypoxia before being depleted for ING1b, HIF1*α*. Subsequently, cells were incubated with adipogenic induction medium under hypoxia. Knockdowns and media changes were performed every 3 days. Adipocyte marker (*FABP4, LPL, PPARγ2*) expressions were analyzed by Q-PCR for day 7 samples. (**b**) Adipogenic differentiation of ADSCs in normoxic conditions. ADSCs were knocked down every 3 days for ING1 and HIF1*α* under normoxic conditions during the adipogenic induction. (**c**) Efficiencies of ING1b and HIF1*α* siRNAs were evaluated at day 7 by Q-PCR. (**d**) No difference in expressions of adipocyte markers is observed following ING1b depletion at the end of 14 days of adipogenic induction, suggesting ING1b is not involved in a same manner during the adipocyte differentiation at 7 and 14 days of the adipogenic induction. *FABP4, LPL* and *PPARγ2* mRNA levels were evaluated by Q-PCR. All mRNA levels were normalized to *U6* expression. Bars represent mean±S.E.M. *P*<0.05 (*), *P*<0.01 (**), *P*<0.001 (***), non significant (NS) in an unpaired Student's *t*-test (*n*=3)

HIF1*α* has been described to reduce mesenchymal stromal cell commitment to adipocyte differentiation.^23^ Therefore, we hypothesized that the lack of ING1b in hypoxic context could increase HIF1*α* levels, and subsequently impair differentiation processes. To verify this hypothesis, we knocked down ING1b expression and induced adipocyte differentiation. We observed that ADSCs depleted for ING1b could not express as much peroxisome proliferator-activated receptor *γ*2 (PPAR*γ*2), lipoprotein lipase (LPL) or fatty acid binding protein 4 (FABP4), as their respective controls, after 7 days of adipogenic induction (Figure 5a). Conversely, HIF1*α*-depleted cells expressed higher rates of these three adipocyte markers. This argues that ING1b takes part in adipocyte markers expressions while HIF1*α* prevents them. When these experiments lasted up to 14 days, we observed that adipogenesis still occurred after day 7, as judging by increasing rates of late adipocyte markers, that is, *FABP4* and *LPL*. However, no significant difference was observed for adipocyte markers expression between control and ING1b-depleted cells (Figure 5d). Adipogenic induction experiments were performed under normoxic conditions, a context more prone to adipocyte differentiation until day 7. We observed that this differentiation process was more efficient in normoxia and, by contrast to hypoxic conditions, ING1 silencing did not impair the adipocyte differentiation (Figure 5b,Supplementary Figure 6). These findings suggest that during early adipogenic differentiation, hypoxia mediated a HIF1*α*-dependent decrease of adipocyte commitment, and conversely ING1b specifically favors expressions of adipocyte markers. Interestingly, the concomitant silencing of ING1b and HIF1*α* led to normal expression of adipocyte markers (Figure 5a). This suggests that ING1b and HIF1*α* may have opposite effects on the early steps of adipogenic induction. Besides, after these 7 days of adipogenic induction, we observed that the expression of delta-like homolog 1/preadipocyte factor-1 (DLK1/PREF-1) expression decreased in all the tested cases, suggesting a loss in the mesenchymal character of ADSCs (Supplementary Figure 5) and an efficient induction of adipogenesis.^24^

## Discussion

Long-term cultures expose MSCs to replicative stress or DNA damage, which may result in a loss of their multipotency and immunomodulatory properties, genomic instability and eventually senescence.^25, 26, 27^ However, most of these studies have relied on culture protocols using normoxia (21% O_2_). Culture of MSCs under hypoxic conditions, reproducing hypoxic niches, has been suggested as a way to maintain their immature phenotype^28^ and to delay senescence. However, hypoxia has also been demonstrated to induce a form of replicative stress (<0.1% O_2_)^29^ and to hamper expression of homologous recombination factors (Rad51 and BRCA1) in cancer cell lines, BM-MSCs and in ADSCs.^30, 31, 32^

In this work, we first intended to assess whether ADSCs were able to preserve their genomic integrity and decided to evaluate possible roles of the ING1 protein. First, we observed that following doxorubicin treatments in hypoxia (1% O_2_), only ADSCs (‘population doubling level'<20) could increase their ING1b protein levels, compared with U2OS cells. These results are consistent with previous studies emphasizing that the regulation of ING1b by DNA damage is not general, and may be cell type or stimulus specific.^14, 33, 34^

**Figure 6:**
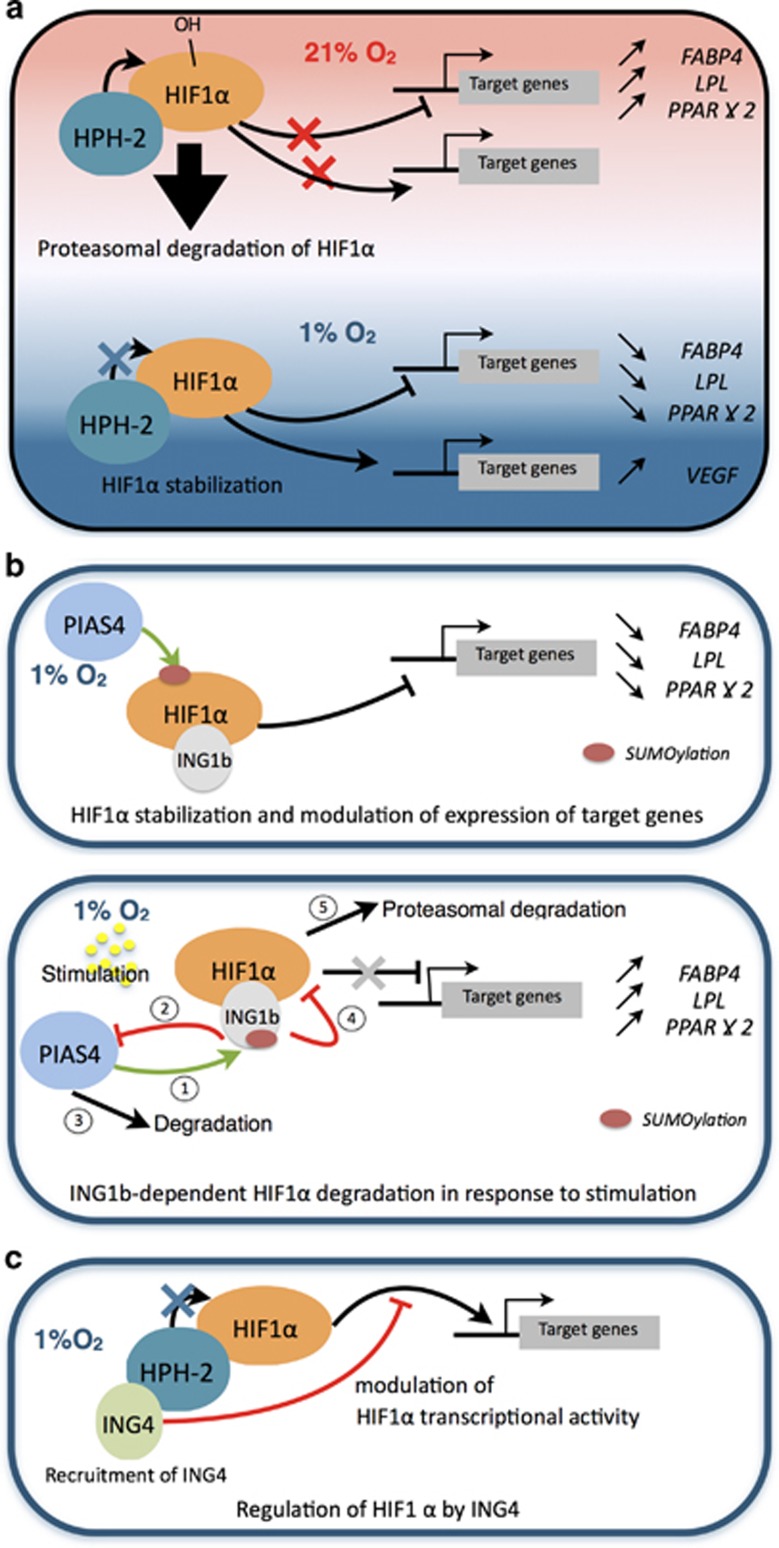
A model for ING1b-dependent degradation of HIF1*α* in hypoxia. (**a**) HIF1*α* regulation in normoxic and hypoxic conditions. Under normoxic conditions HIF1*α* interact with HIF prolyl hydroxylase 2 (HPH-2) and is degraded. Under hypoxic conditions, hydroxylation of HIF1*α* by HPH-2 is impaired, resulting in its stabilization. (**b**) In hypoxia in ADSCs or U2OS cells, PIAS4 stabilizes HIF1*α* in presence or absence of ING1b and HIF1*α* impairs expression of adipocyte markers when adipogenesis is induced (upper panel). Following stimulation (doxorubicin treatment or adipogenic induction), ING1b is stabilized in a PIAS4-dependent manner (lower panel). PIAS4 is then degraded in an ING1b-dependent manner and ING1b subsequently promotes HIF1*α* proteasomal degradation and the expression of adipocyte markers. (**c**) Regulation of HIF1*α* by ING proteins. Under hypoxic conditions, ING4 has been demonstrated to bind the HIF1*α* protein complex through interactions with HPH-2, thus preventing the transcription activity of HIF1*α*

We observed a decrease of HIF1*α* levels in ADSCs as a result of an ING1b accumulation upon doxorubicin treatment. Conversely, HIF1*α* levels increased in absence of ING1b. Additional experiments revealed that HIF1*α* destabilization by ING1b occurred at a protein level. This was also observed in U2OS cells cultured in hypoxia with lowered HIF1*α* protein levels in an ING1b-dependent manner. We show ING1b interacts with HIF1*α* and promotes its degradation. ING1b is known to prevent genomic stability through chromatin remodeling and cell cycle arrest^34, 35, 36^ in response to DNA damage. ING1b as other ING proteins (ING2), is involved in the regulation of replicative fork progression, particularly in lesion bypass.^37, 38^ Our data suggest that increasing ING1b levels, observed in ADSCs following doxorubicin treatment, could decrease the HIF1*α* protein levels, and consequently the hypoxic response. Thus, ING1b may prevent the hypoxia-mediated genomic instability by opposing to the HIF1*α*-mediated transcriptional downregulation of proteins involved in DNA repair.^39, 40^ Nonetheless, the hypoxic response may be regulated at different levels by different ING proteins. A previous study demonstrated that HIF activities could be modulated by ING4^12^ (Figure 6e). Indeed, under hypoxic conditions, stabilized HIF factors, which still interact with HPH-2, allow the recruitment of ING4 to the promoters of HIF target genes eventually preventing their expressions. Here, the plant homeodomain of ING4 seemed required for its interactions with HPH-2. Usually described to recognize H3K4me2 or H3K4me3, the PHD appears to bind other proteins, like HPH-2 or p65 in the case of ING4.^41^ Therefore, an investigation on the ING1b PHD remains to be done for the comprehension of the regulation of HIF1*α*, and eventually its degradation. Besides, because this PHD is a common feature of the ING proteins, this suggests that other ING proteins: ING2, ING3 and ING5 could play a role in the hypoxic response as well.

We noticed that ING1b, when mutated on its SUMOylation site, failed to trigger HIF1*α* degradation, suggesting that the K193 SUMOylation site (mono or diSUMOylation) is required for these effects. In addition, the E3 ligase PIAS4, involved in ING1b SUMOylation seemed to be required for ING1b increase following genotoxic stress.^42^ Our results also support the study of Ceruti *et al.*,^34^ claiming that disconnects between ING1 mRNA and protein levels following stimuli, like DNA damage, depend on the existence of additional cellular mechanisms that modulate ING1b levels. Interestingly, PIAS4 level was regulated in an ING1b-dependent manner. Indeed, ING1b silencing was accompanied by increasing levels of PIAS4 in ADSCs. Taken together, our results argue, at least under hypoxia, PIAS4 and ING1b can regulate each other's activities. Moreover, ING1b may, through modulation of PIAS4 activities, finely regulate HIF1*α* whose protein levels appeared to rely on PIAS4 as well. Our results confirm that post-translational modifications like SUMOylation (mono or diSUMOylation), clearly modulate ING1 activities,^14, 43^ and suggest that SUMOylation might be responsible for a shift from a conserved ING1b/HIF1*α* complex to HIF1*α* degradation. This indicates that ING1b SUMOylation may alter interactions with HIF1*α*, either by masking binding sites or by a global conformational change.^44^ Overall, we showed that SUMOylation of ING1b, critical for HIF1*α* degradation, might require proteins modulating SUMOylation, as PIAS4, or deSUMOylation processes.

The hypoxic response and HIF1*α* are critical for the maintenance of MSC properties and their immature state,^4, 45, 46^ and the roles of ING proteins have been minimally studied in MSCs. Nonetheless, it seemed conceivable that ING1b, because of its functions could be involved in MSC fate. Adipogenesis has been frequently associated with mid to high oxygen tensions. Indeed, markers of adipocytes, like LPL or PPAR*γ*2, have been shown to be reduced following HIF1*α* activation.^47, 48^ As a consequence, we hypothesized that ING1b, by reducing HIF1*α* protein levels could facilitate this differentiation process. In agreement with our hypothesis, we observed that the presence of ING1b was required for expression of these adipocyte markers during the early/intermediate days of the adipogenesis process (day 7), whereas HIF1*α* was associated to their downregulations. Thus, we suggested that ING1b might be involved in the adipocyte differentiation, by decreasing the level of HIF1*α* in hypoxia. However, our results do not exclude the possibility that ING1b could have more roles in cell differentiation. Cheng *et al.*^49^ showed that ING1, as a reader of H3K4me3 histone marks, was needed for myoblast differentiation by promoting the expression of myoblast-specific genes. H3K4me3 marks have been associated with active lineage-specific promoters in ADSCs (*LPL*, *FABP4* and *PPARγ2*) during differentiation, and interestingly HIF1*α* has been reported to target open chromatin regions associated with H3K4me3.^50,51^ Thus, hypoxia may constitutively repress the expression of *LPL*, *FABP4* and *PPARγ2*, through HIF1*α*. In addition, when ING1b and HIF1*α* were both depleted, adipocyte markers were normally expressed in hypoxic conditions, suggesting that adipocyte markers expression were no longer modulated. Thus, as for the genomic stability in hypoxia, ING1b might be involved in differentiation processes at different levels and may act differently according the presence of other transcription factors. We also evaluated the levels of *DLK1/PREF*-1, an early mesenchymal marker,^24^ at the end of 7 days of adipogenic induction in hypoxia. Although our culture context was prone to keep elevated levels of DLH1/Pref-1 with probable elevated levels of HIF1*α* and HIF2*α* proteins,^52^ we noticed that *DLH1/Pref-*1 decreased during this adipogenic induction. Taken together, we can conclude that the hypoxic culture context did not impede the triggering of the adipocyte differentiation. However, critical adipocyte markers like PPAR*γ*2 present at more early stages or later adipocyte markers like LPL and mostly FABP4 have been modulated through the 14 days of adipogenic induction. Thus, we observed that ING1 was required for these adipocyte marker expressions under hypoxia until day 7 of adipogenic induction. Conversely, HIF1*α*, a main marker of the hypoxic response repressed their expressions.

Finally, we propose a mechanism where ING1b may prevent the hypoxic response, either by reducing HIF1*α* protein content in the nucleus or on chromatin. As we report, expressions of adipocyte markers (*LPL*, *FABP4*, *PPARγ2*) were reduced by HIF1*α* in hypoxia (Figures 5a, 6a and b). Our study suggests that HIF1*α* degradation, promoted by ING1b, may increase expression levels of *LPL*, *FABP4* and *PPARγ2* (Figure 6b). As a consequence, ING1b could potentiate the differentiation of ADSCs. Moreover, ING1b can interact with histone modifiers, like SIRT1,^53^ already described to modulate HIF1*α* inactivation and to regulate differentiation of MSCs.^54, 55^ Therefore, ING1b could take part in a complex regulating MSC's fate, especially under low oxygen tension.

In our study, we focused on adipocyte differentiation under hypoxic conditions. Of note, we failed to induce osteogenesis under hypoxia with ADSCs, although other studies have discussed the benefits of hypoxia on osteogenesis^23^ (data not shown). Thus, we could not conclude on a role of ING1b in this context. Nevertheless, we proceeded to osteogenic induction in 21% O_**2**_ (Supplementary Figure 7) and noticed that when ING1 was silenced in normoxic conditions, *RUNX2*, *BGLAP* and *SP7* levels increased, as well as calcium deposits at 7 or 14 days, suggesting that as for adipocyte marker expressions under normoxia, ING1b might decrease osteoblast marker expressions in normoxia as well. Interestingly, even if it challenged the physiological sense, it appeared that HIF1*α* knockdown enhanced osteogenesis in normoxia. This suggests that HIF1*α* might be sufficiently maintained in normoxia for modulating expression of some targets. This is in accordance with the study by Palomäki *et al.*^6^ that demonstrated great levels of HIF1*α* in normoxia in MSCs. Thus, ING1b appeared to display various roles according to the commitment and the culture context of ADSCs.

## Conclusion

So far, ING proteins roles in hypoxia have been little studied. In this study we focused on ING1b in a mesenchymal stromal cell model, whose properties are particularly dependent on oxygen tension. As depicted in the Figure 6b, ING1b, under the control of its SUMOylation status appears to regulate the hypoxic response by triggering the proteasomal degradation of HIF1*α*, which eventually could lead to changes in its target genes expression (VEGF and EPO). Nonetheless, the hypoxic response may be regulated at different levels (Figure 6c) by other ING proteins, as previous study demonstrated with ING4.^12^

## Materials and Methods

### Culture of ADSCs and induction of adipogenesis

Healthy donor recruitment followed the institutional review board approval and written informed consent process according to the Declaration of Helsinki. The stromal vascular fraction (SVF) was isolated from human abdominal lipoaspirates of adults undergoing reconstructive surgery after weight loss.^56^ Lipoaspirates were centrifuged for 5 min at 600 × *g*. Phases containing adipocyte debris and red blood cells were removed. Tissues were digested with type IV collagenase (200 U/ml), neutral protease dispase (1.6 U/ml; Worthington, Freehold, NJ, USA) and DNAse (Pulmozyme,10 U/ml, Roche, Neuilly-sur-Seine, France) for 45 min at 37 °C with gentle rocking. Lysates were passed through a 100 *μ*m cell strainer and centrifuged for 10 min at 600 × *g* to obtain the SVF. Cells from the SVF were seeded at 1000 cells/cm^2^ in *α*MEM supplemented with 10% selected fetal calf serum (HyClone, FCS, Thermo Scientific, Villebon sur Yvette, France), 1 ng/ml of bFGF (Cellgenix, Clermont Ferrand, France), penicillin (100 U/ml), and streptomycin (100 *μ*g/ml; Life Technologies). ADSCs were analyzed for CD73, CD90, CD45 and CD31 presences at the end of the first passage (Supplementary Figure 1). The established ADSC primary lines were passaged at 90% of confluence and plated at 2000–2500 cells/cm^2^ and cultivated in *α*MEM supplemented with 10% selected Hyclone FCS, 1 ng/ml of bFGF, penicillin (100 U/ml), and streptomycin (100 *μ*g/ml). ADSCs from three different donors were used in experiments between 15 and 20 cumulative population doublings.

Adipogenesis induction was performed using the hMSC Mesenchymal Stem Cell Adipogenic Differentiation Medium (Lonza, Verviers, Belgium) according to the manufacturer's recommendations. ADSCs were cultured at 37 °C, 5% CO_2_ and 21 or 1% O_2_, in agreement with the experiment purposes. The medium was changed every 3 days and the differentiation period lasted 7 or 14 days.

U2OS oesteosarcoma cells (p53^+/+^) were grown in McCoy's 5A modified medium supplemented with 10% FCS (Life Technologies), penicillin (100 U/ml) and streptomycin (100 *μ*g/ml).

Cells were amplified in normoxia (5% CO_2_, 21% O_2_) at 37 °C and placed in a Binder CB incubator (VWR International, Fontenay-sous-Bois, France) for a 24 h buffering time before each experiment under hypoxic conditions (5% CO_2_, 1% O_2_) at 37 °C.

### Transfections

Silencing experiments were performed with Lipofectamine RNAiMAX Transfection Reagent (Life Technologies) according to the manufacturer's instructions. ING1b, PIAS4 stealth RNAi siRNAs (Life Technologies) and HIF1*α* siRNA (SC-35561, Santa Cruz Biotechnology, CliniSciences, Nanterre, France) were used in experiments. A Stealth RNAi siRNA Negative Control (Life Technologies) was used as control.

Stably transfected U2OS cells were previously produced using the empty pcDNA 3.1 vector, pcDNA 3.1 ING1b WT (coding for ING1b) and pcDNA 3.1 ING1b E195 and selected with G148 (0.5 mg/ml).^15^

Transient transfections with empty pcDNA 3.1 (control), pcDNA 3.1 Flag ING1b WT plasmids were performed on ADSCs using a Human MSC Nucleofector Kit and the Nucleofector device (Lonza).

### Western blot and immunoprecipitations experiments

Protein samples were obtained after washing cells in cold 1X PBS buffer and harvesting in RIPA buffer containing a protease inhibitor cocktail (Roche). Supernatants were collected after a 4 °C centrifugation at 16 000 *× g* for 15 min. Samples were subjected to electrophoresis using the NuPage Novex Bis-Tris Gel Electrophoresis system (Life Technologies), and transferred to nitrocellulose membrane. The antibodies used were anti-PIAS4, anti-HIF1*α*, anti-ING1b (Cab3) (Santa Cruz Biotechnology), anti-*β*actin (Sigma, St Quentin Fallawier, France) and anti-γH2AX (Cell Signaling).

After 24 h of incubation under hypoxic conditions, cells were washed in cold 1X PBS buffer and harvested in IP lysis buffer (Thermo Fisher Scientific, Villebon sur Yvette, France), containing a protease inhibitor cocktail. Lysates were centrifuged at 4 °C for 15 min at 16 000 *× g* and supernatants were recovered. Equal amounts of protein extracts were incubated with the ING1b (Cab1 and Cab5) or HIF1*α* antibodies for 3 h and then respectively bound to Dynabeads Protein G and protein A (Life Technologies) at 4 °C with rocking. Beads were washed three times with cold 1X PBS buffer+0.01% Tween20. Immunoprecipitated samples were analyzed by Western blot.

Western blots pictures were obtained with a Gbox imager (Syngene, Cambridge, UK) and were quantified using ImageJ software (U. S. National Institutes of Health, Bethesda, MD, USA, http://imagej.nih.gov/ij/). Results display the amount of protein of interest normalized on actin amount. Data are represented as percentages.

### Quantitative RT-PCR

RNA was extracted using the RNeasy Kit (Qiagen, Courtaboeuf, France) and cDNAs were generated using the Superscript II reverse transcriptase (Life Technologies). RT-PCR amplification experiments were performed with the SYBR Green PCR Master Mix on a StepOnePlus Real-Time PCR System (Applied Biosystems, Life Technologies, Villebon sur Yvette, France). The relative gene expression was calculated with the 2^−^^ΔΔCT^ method. All the results were normalized to *U6* expression (similar results were obtained for housekeeping genes such as *RPL13a*, not shown). The primer sequences used in RT-PCR are listed in Supplementary Figure 8.

### Statistical analyzes

ADSC PCR data is presented as mean±S.E.M. The statistical significance was determined using an unpaired Student's *t*-test. Values of *P*<0.05 (*), *P*<0.01 (**) and *P*<0.001 (***) were considered statistically significant. Experiments were repeated three times.

## Abbreviations

HIF1*α*: hypoxia inducible factor 1 alpha
ING: inhibitor of growth
SUMO: small ubiquitin-like modifier
PIAS4: protein inhibitor of activated STAT protein *γ*
ADSC: adipose-derived stromal cell

## References

[bib1] Uccelli A, Moretta L, Pistoia V. Mesenchymal stem cells in health and disease. Nat Rev Immunol 2008; 8: 726–736. 19172693 10.1038/nri2395

[bib2] Ménard C, Tarte K. Immunoregulatory properties of clinical grade mesenchymal stromal cells: evidence, uncertainties, and clinical application. Stem Cell Res Ther 2013; 4: 64. 23742637 10.1186/scrt214 PMC3706914

[bib3] Wagner W, Horn P, Castoldi M, Diehlmann A, Bork S, Saffrich R et al. Replicative senescence of mesenchymal stem cells: a continuous and organized process. PLoS One 2008; 3: e2213. 18493317 10.1371/journal.pone.0002213 PMC2374903

[bib4] Fehrer C, Brunauer R, Laschober G, Unterluggauer H, Reitinger S, Kloss F et al. Reduced oxygen tension attenuates differentiation capacity of human mesenchymal stem cells and prolongs their lifespan. Aging Cell 2007; 6: 745–757. 17925003 10.1111/j.1474-9726.2007.00336.x

[bib5] Mohyeldin A, Garzón-Muvdi T, Quiñones-Hinojosa A. Oxygen in stem cell biology: a critical component of the stem cell niche. Cell Stem Cell 2010; 7: 150–161. 20682444 10.1016/j.stem.2010.07.007

[bib6] Palomäki S, Pietilä M, Laitinen S, Pesälä J, Sormunen R, Lehenkari P et al. HIF-1*α* is upregulated in human mesenchymal stem cells. Stem Cells 2013; 31: 1902–1909. 23744828 10.1002/stem.1435

[bib7] Tsai CC, Chen YJ, Yew TL, Chen LL, Wang JY, Chiu CH et al. Hypoxia inhibits senescence and maintains mesenchymal stem cell properties through down-regulation of E2A-p21 by HIF-TWIST. Blood 2011; 117: 459–469. 20952688 10.1182/blood-2010-05-287508

[bib8] Guérillon C, Larrieu D, Pedeux R. ING1 and ING2: multifaceted tumor suppressor genes. Cell Mol Life Sci 2013; 70: 3753–3772. 23412501 10.1007/s00018-013-1270-z PMC11113716

[bib9] Tallen G, Farhangi S, Tamannai M, Holtkamp N, Mangoldt D, Shah S et al. The inhibitor of growth 1 (ING1) proteins suppress angiogenesis and differentially regulate angiopoietin expression in glioblastoma cells. Oncol Res 2009; 18: 95–105. 20066899 10.3727/096504009789954645

[bib10] Blagosklonny MV, An WG, Romanova LY, Trepel J, Fojo T, Neckers L. p53 inhibits hypoxia-inducible factor-stimulated transcription. J Biol Chem 1998; 273: 11995–11998. 9575138 10.1074/jbc.273.20.11995

[bib11] Kaluzová M, Kaluz S, Lerman M I, Stanbridge E J. DNA damage is a prerequisite for p53-mediated proteasomal degradation of HIF-1alpha in hypoxic cells and downregulation of the hypoxia marker carbonic anhydrase IX. Mol Cell Biol 2004; 24: 5757–5766. 15199132 10.1128/MCB.24.13.5757-5766.2004 PMC480909

[bib12] Ozer A, Wu L C, Bruick R K. The candidate tumor suppressor ING4 represses activation of the hypoxia inducible factor (HIF). Proc Natl Acad Sci USA 2005; 102: 7481–7486. 15897452 10.1073/pnas.0502716102 PMC1140452

[bib13] Strioga M, Viswanathan S, Darinskas A, Slaby O, Michalek J. Same or not the same? Comparison of adipose tissue-derived versus bone marrow-derived mesenchymal stem and stromal cells. Stem Cells Dev 2012; 21: 2724–2752. 22468918 10.1089/scd.2011.0722

[bib14] Garate M, Campos E I, Bush J A, Xiao H, Li G. Phosphorylation of the tumor suppressor p33(ING1b) at Ser-126 influences its protein stability and proliferation of melanoma cells. FASEB J 2007; 21: 3705–3716. 17585055 10.1096/fj.07-8069com

[bib15] Satpathy S, Guérillon C, Kim TS, Bigot N, Thakur S, Bonni S et al. SUMOylation of the ING1b tumour suppressor regulates gene transcription. Carcinogenesis 2014; 35: 2214–2223. 24903338 10.1093/carcin/bgu126 PMC4178466

[bib16] Carbia-Nagashima A, Gerez J, Perez-Castro C, Paez-Pereda M, Silberstein S, Stalla GK et al. RSUME, a small RWD-containing protein, enhances SUMO conjugation and stabilizes HIF-1alpha during hypoxia. Cell 2007; 131: 309–323. 17956732 10.1016/j.cell.2007.07.044

[bib17] Cheng J, Kang X, Zhang S, Yeh E T H. SUMO-specific protease 1 is essential for stabilization of HIF1alpha during hypoxia. Cell 2007; 131: 584–595. 17981124 10.1016/j.cell.2007.08.045 PMC2128732

[bib18] Bae SH, Jeong JW, Park JA, Kim SH, Bae MK, Choi SJ et al. Sumoylation increases HIF-1alpha stability and its transcriptional activity. Biochem Biophys Res Commun 2004; 324: 394–400. 15465032 10.1016/j.bbrc.2004.09.068

[bib19] Mabb A M, Wuerzberger-Davis S M, Miyamoto S. PIASy mediates NEMO sumoylation and NF-kappaB activation in response to genotoxic stress. Nat Cell Biol 2006; 8: 986–993. 16906147 10.1038/ncb1458

[bib20] Cai Q, Verma S C, Kumar P, Ma M, Robertson E S. Hypoxia inactivates the VHL tumor suppressor through PIASy-mediated SUMO modification. PLoS One 2010; 5: e9720. 20300531 10.1371/journal.pone.0009720 PMC2838797

[bib21] Li J, Xu Y, Long XD, Wang W, Jiao HK, Mei Z et al. Cbx4 governs HIF-1*α* to potentiate angiogenesis of hepatocellular carcinoma by its SUMO E3 ligase activity. Cancer Cell 2014; 25: 118–131. 24434214 10.1016/j.ccr.2013.12.008

[bib22] Kang X, Li J, Zou Y, Yi J, Zhang H, Cao M et al. PIASy stimulates HIF1*α* SUMOylation and negatively regulates HIF1*α* activity in response to hypoxia. Oncogene 2010; 29: 5568–5578. 20661221 10.1038/onc.2010.297

[bib23] Wagegg M, Gaber T, Lohanatha FL, Hahne M, Strehl C, Fangradt M et al. Hypoxia promotes osteogenesis but suppresses adipogenesis of human mesenchymal stromal cells in a hypoxia-inducible factor-1 dependent manner. PLoS One 2012; 7: e46483. 23029528 10.1371/journal.pone.0046483 PMC3459928

[bib24] Hudak CS, Gulyaeva O, Wang Y, Park SM, Lee L, Kang C et al. Pref-1 marks very early mesenchymal precursors required for adipose tissue development and expansion. Cell Rep 2014; 8: 678–687. 25088414 10.1016/j.celrep.2014.06.060 PMC4138044

[bib25] Li XY, Ding J, Zheng ZH, Li XY, Wu ZB, Zhu P. Long-term culture *in vitro* impairs the immunosuppressive activity of mesenchymal stem cells on T cells. Mol Med Rep 2012; 6: 1183–1189. 22923041 10.3892/mmr.2012.1039

[bib26] Wang Y, Zhang Z, Chi Y, Zhang Q, Xu F, Yang Z et al. Long-term cultured mesenchymal stem cells frequently develop genomic mutations but do not undergo malignant transformation. Cell Death Dis 2013; 4: e950. 24309937 10.1038/cddis.2013.480 PMC3877551

[bib27] Estrada JC, Torres Y, Benguría A, Dopazo A, Roche E, Carrera-Quintanar L et al. Human mesenchymal stem cell-replicative senescence and oxidative stress are closely linked to aneuploidy. Cell Death Dis 2013; 4: e691. 23807220 10.1038/cddis.2013.211 PMC3702285

[bib28] Grayson W L, Zhao F, Izadpanah R, Bunnell B, Ma T. Effects of hypoxia on human mesenchymal stem cell expansion and plasticity in 3D constructs. J Cell Physiol 2006; 207: 331–339. 16331674 10.1002/jcp.20571

[bib29] Olcina M M, Foskolou I P, Anbalagan S, Senra J M. Replication stress and chromatin context link ATM activation to a role in DNA replication. Mol Cell 2013; 52: 758–766. 24268576 10.1016/j.molcel.2013.10.019 PMC3898930

[bib30] Bindra RS, Gibson SL, Meng A, Westermark U, Jasin M, Pierce AJ et al. Hypoxia-induced down-regulation of BRCA1 expression by E2Fs. Cancer Res 2005; 65: 11597–11604. 16357170 10.1158/0008-5472.CAN-05-2119

[bib31] Bindra R S, Glazer P M. Repression of RAD51 gene expression by E2F4/p130 complexes in hypoxia. Oncogene 2007; 26: 2048–2057. 17001309 10.1038/sj.onc.1210001

[bib32] Oliveira PH, Boura JS, Abecasis MM, Gimble JM, da Silva CL, Cabral JM. Impact of hypoxia and long-term cultivation on the genomic stability and mitochondrial performance of *ex vivo* expanded human stem/stromal cells. Stem Cell Res 2012; 9: 225–236. 22903042 10.1016/j.scr.2012.07.001

[bib33] Rogakou E P, Boon C, Redon C, Bonner W M. Megabase chromatin domains involved in DNA double-strand breaks *in vivo*. J Cell Biol 1999; 146: 905–916. 10477747 10.1083/jcb.146.5.905 PMC2169482

[bib34] Ceruti J M, Ogara M F, Menéndez C, Palmero I, Cánepa E T. Inhibitor of growth 1 (ING1) acts at early steps of multiple DNA repair pathways. Mol Cell Biochem 2013; 378: 117–126. 23459830 10.1007/s11010-013-1601-2

[bib35] Cheung K J, Mitchell D, Lin P, Li G. The tumor suppressor candidate p33(ING1) mediates repair of UV-damaged DNA. Cancer Res 2001; 61: 4974–4977. 11431327

[bib36] Peña PV, Hom RA, Hung T, Lin H, Kuo AJ, Wong RP et al. Histone H3K4me3 binding is required for the DNA repair and apoptotic activities of ING1 tumor suppressor. J Mol Biol 2008; 380: 303–312. 18533182 10.1016/j.jmb.2008.04.061 PMC2576750

[bib37] Larrieu D, Ythier D, Binet R, Brambilla C, Brambilla E, Sengupta S et al. ING2 controls the progression of DNA replication forks to maintain genome stability. EMBO Rep 2009; 10: 1168–1174. 19730436 10.1038/embor.2009.180 PMC2759735

[bib38] Wong RP, Lin H, Khosravi S, Piche B, Jafarnejad SM, Chen DW et al. Tumour suppressor ING1b maintains genomic stability upon replication stress. Nucleic Acids Res 2011; 39: 3632–3642. 21227930 10.1093/nar/gkq1337 PMC3089469

[bib39] Koshiji M, To KK, Hammer S, Kumamoto K, Harris AL, Modrich P et al. HIF-1alpha induces genetic instability by transcriptionally downregulating MutSalpha expression. Mol Cell 2005; 17: 793–803. 15780936 10.1016/j.molcel.2005.02.015

[bib40] Rodríguez-Jiménez F J, Moreno-Manzano V, Lucas-Dominguez R, Sánchez-Puelles J-M. Hypoxia causes downregulation of mismatch repair system and genomic instability in stem cells. Stem Cells 2008; 26: 2052–2062. 18511603 10.1634/stemcells.2007-1016

[bib41] Hou Y, Zhang Z, Xu Q, Wang H, Xu Y, Chen K. Inhibitor of growth 4 induces NFκB/p65 ubiquitin-dependent degradation. Oncogene 2013; 33: 1997–2003. 23624912 10.1038/onc.2013.135

[bib42] Galanty Y, Belotserkovskaya R, Coates J, Polo S, Miller KM, Jackson SP. Mammalian SUMO E3-ligases PIAS1 and PIAS4 promote responses to DNA double-strand breaks. Nature 2009; 462: 935–939. 20016603 10.1038/nature08657 PMC2904806

[bib43] Yu L, Thakur S, Leong-Quong RY, Suzuki K, Pang A, Bjorge JD et al. Src regulates the activity of the ING1 tumor suppressor. PLoS One 2013; 8: e60943. 23585863 10.1371/journal.pone.0060943 PMC3621671

[bib44] Geiss-Friedlander R, Melchior F. Concepts in sumoylation: a decade on. Nat Rev Mol Cell Biol 2007; 8: 947–956. 18000527 10.1038/nrm2293

[bib45] Lee J, Jung E, Hyun J W, Park D. Ultraviolet A regulates the stemness of human adipose tissue-derived mesenchymal stem cells through downregulation of the HIF-1*α* via activation of PGE(2)-cAMP signaling. J Cell Biochem 2012; 113: 3681–3691. 22753248 10.1002/jcb.24241

[bib46] Mathieu J, Zhang Z, Nelson A, Lamba DA, Reh TA, Ware C et al. Hypoxia induces re-entry of committed cells into pluripotency. Stem Cells 2013; 31: 1737–1748. 23765801 10.1002/stem.1446 PMC3921075

[bib47] Yun Z, Maecker H L, Johnson R S, Giaccia A J. Inhibition of PPAR gamma 2 gene expression by the HIF-1-regulated gene DEC1/Stra13: a mechanism for regulation of adipogenesis by hypoxia. Dev Cell 2002; 2: 331–341. 11879638 10.1016/s1534-5807(02)00131-4

[bib48] Zhou S, Lechpammer S, Greenberger J S, Glowacki J. Hypoxia inhibition of adipocytogenesis in human bone marrow stromal cells requires transforming growth factor-beta/Smad3 signaling. J Biol Chem 2005; 280: 22688–22696. 15845540 10.1074/jbc.M412953200 PMC1242109

[bib49] Cheng J, Blum R, Bowman C, Hu D, Shilatifard A, Shen S et al. A role for H3K4 monomethylation in gene repression and partitioning of chromatin readers. Mol Cell 2014; 53: 979–992. 24656132 10.1016/j.molcel.2014.02.032 PMC4031464

[bib50] Xia X, Kung A L. Preferential binding of HIF-1 to transcriptionally active loci determines cell-type specific response to hypoxia. Genome Biol 2009; 10: R113. 19828020 10.1186/gb-2009-10-10-r113 PMC2784328

[bib51] Noer A, Lindeman L C, Collas P. Histone H3 modifications associated with differentiation and long-term culture of mesenchymal adipose stem cells. Stem Cells Dev 2009; 18: 725–736. 18771397 10.1089/scd.2008.0189

[bib52] Kim Y, Lin Q, Zelterman D, Yun Z. Hypoxia-regulated delta-like 1 homologue enhances cancer cell stemness and tumorigenicity. Cancer Res 2009; 69: 9271–9280. 19934310 10.1158/0008-5472.CAN-09-1605 PMC2828615

[bib53] Binda O, Nassif C, Branton P E. SIRT1 negatively regulates HDAC1-dependent transcriptional repression by the RBP1 family of proteins. Oncogene 2008; 27: 3384–3392. 18193082 10.1038/sj.onc.1211014

[bib54] Lim JH, Lee YM, Chun YS, Chen J, Kim JE, Park JW. Sirtuin 1 modulates cellular responses to hypoxia by deacetylating hypoxia-inducible factor 1alpha. Mol Cell 2010; 38: 864–878. 20620956 10.1016/j.molcel.2010.05.023

[bib55] Simic P, Zainabadi K, Bell E, Sykes DB, Saez B, Lotinun S et al. SIRT1 regulates differentiation of mesenchymal stem cells by deacetylating *β*-catenin. EMBO Mol Med 2013; 5: 430–440. 23364955 10.1002/emmm.201201606 PMC3598082

[bib56] Gil-Ortega M, Garidou L, Barreau C, Maumus M, Breasson L, Tavernier G et al. Native adipose stromal cells (ASCs) egress from adipose tissue *in vivo*: evidence during lymph node activation. Stem Cells 2013; 31: 1309–1320. 23533182 10.1002/stem.1375

