# Supplementary material for: ING1b negatively regulates HIF1α protein levels in adipose-derived stromal cells by a SUMOylation-dependent mechanism
Source: Cell Death Dis. 2015 Jan 22;6(1):e1612–. doi: 10.1038/cddis.2014.577 (PMC4669774; doi:10.1038/cddis.2014.577)
Supplement: Supplementary Information [file cddis2014577x1.pdf]

Table of Contents:

Supplementary Materials and methods

Supplementary Figure 1. Characterization of adipose derived stromal cells (ADSCs).

Supplementary Figure 2. ING1b depletion in ADSCs.

Supplementary Figure 3. SUMOylation of ING1b in ADSCs in hypoxia.

Supplementary Figure 4. Comparison of ING1b protein levels in ADSCs and U2OS in hypoxia.

Supplementary Figure 5. *DLK1* expression at 7 days of adipogenic differentiation in hypoxia.

Supplementary Figure 6. Adipogenic induction under 21% O_2 after 7 and 14 days.

Supplementary Figure 7. Osteogenic induction under 21% O_2 after 7 and 14 days.

Supplementary Figure 8. Sequences of primers used in the study.

Supplementary Materials and methods

SUMOylation experiments

ADSCs were cultured under 1% O_2 for 24 hours before transfection. Cells were transfected under 1% O_2 with pCMV6-Flag-SUMO1 and pcDNA 3.1 ING1b WT; or with the empty pCMV6 vector using the viromer® yellow reagent (Lipocalyx) and incubated for 24 supplemental hours in hypoxia. Cell were washed in cold 1X PBS buffer and harvested in RIPA buffer containing a protease inhibitor cocktail and *N*-Ethylmaleimide to prevent de-SUMOylation events (20 mM). Lysates were heated (2 minutes, 98°C), sonicated and centrifuged for 15 minutes at 16,000g (4°C). Supernatants were incubated with anti-FLAG® M2 affinity gel (Sigma) for 4 hours at 4°C with rocking. Beads were washed three times with cold 1X PBS buffer + 0.01% Tween20. Immunoprecipitated samples were analyzed by western blot.

Osteogenis induction

Osteogenesis induction was performed using the hMSC Mesenchymal Stem Cell Osteogenic Differentiation Medium (Lonza) according to the manufacturer's recommendations. ADSCs were cultured at 37°C, 5% CO_2 and 21% O_2, in agreement with the experiment purposes. The medium was changed every three days and the differentiation period lasted 14 days.

Supplementary Figure 1 Characterization of adipose derived stromal cells (ADSCs) at P1. A representative phenotype profile of ADSCs stained for CD45, CD31, CD73 and CD90 (grey). The isotyped matched controls are shown in black. The ratio between specific antibody and isotype control arithmetic mean fluorescence intensity are indicated in top right corners. ADSCs were analyzed on a Navios cytometer for CD73, CD90, CD45 and CD31 presences at the end of the first passage with CD45 PE (Beckman Coulter), CD31 PerCP eFluor 710 (eBioscience), CD73 PE and CD90 PE (BD Biosciences) and their isotyped matched controls IgG1 PE (BD Biosciences) or IgG1 PerCP eFluor 710 (eBioscience).

Supplementary Figure 2 ING1b depletion in ADSCs. ADSCs cultured in for 24 hours in hypoxia were transfected with ING1 targeting siRNA. Cells were incubated 48 hours later with doxorubicin (10 µM) Dox for 3 hours and medium was changed. At the end of 72 hours of ING1b down regulation, cells were harvested and whole-cell lysates used in Western blot experiments with anti-ING4 (Santa Cruz Biotechnology) and anti-βactin (Sigma) antibodies.

Supplementary Figure 3 SUMOylation of ING1b in ADSCs in hypoxia. ADSCs were cultured for 24 hours in hypoxia before transfection with pCMV6-Flag-SUMO1 (SUMO1) and pcDNA 3.1 ING1 WT (ING1b WT) or pCMV6 (control). Twenty four hours later, cells were harvested and whole-cell extracts were used in a Flag immunoprecipitation. Samples were analyzed for ING1b and βactin. The upper and lower bands in the IP flag section respectively represent the diSUMOylated and monoSUMOylated form of ING1b. Input represent the amount of ING1b in ADSCs after transfection.

Supplementary Figure 4 Comparison of ING1b protein levels in ADSCs and U2OS in hypoxia. ADSCs and U2OS cells were cultured for 24 hours in hypoxia before being transfected with ING1 targeting siRNA. Forty eight hours later, cells were treated with doxorubicin (10 µM) Dox for 3 hours and media were changed. At the end of 72 hours of ING1b down regulation, cells were harvested and whole-cell lysates were used in Western blot experiments. Samples were analyzed for ING1 (Cab 3), ɣH2AX (Cell Signaling) and βactin (Sigma). Two exposures were shown for ING1b and PIAS4 to display the different protein levels between the two cell types.

Supplementary Figure 5 *DLK1* expression at 7 days of adipogenic differentiation in hypoxia. ADSCs were submitted to adipogenic differentiation for 7 days during ING1b and HIF1α knockdowns experiments experiments under hypoxic conditions. The mRNA levels of the early mesenchymal marker *DLK1* were assayed by qRT-PCR. The adipogenic induction in hypoxia was effective and decreased *DLK1* expression. Bars represent means ± SEM. $P<0.01$ (**), $P<0.001$ (***), in Student's t-test (n=3).

Supplementary Figure 6 ADSCs were cultured under 21% O_2 and submitted to an adipogenic differentiation upon ING1 and/or HIF1α knockdowns for 14 days (SCR: control siRNA). Intracellular lipid accumulation was assayed with an Oil Red O staining of lipid vacuoles. Representative pictures taken at 7 and 14 days are presented. Scale bars= 50 µm.

Supplementary Figure 7 ADSCs cultured under 21% O_2 were submitted to an osteogenic induction upon ING1 and/or HIF1α knockdowns. (a) Osteoblast markers (*RUNX2*, *BGLAP*, *SP7*) were analyzed after 7 days of osteogenic induction. Bars represent means ± SEM. $P<0.05$ (*), $P<0.01$ (**), in Student's t-test (n=2). (*b*) Osteoblast specific calcium deposits were revealed with an Alizarin Red staining at day 7 and day 14 of the osteogenic induction under 21% O_2. A representative experiment is presented. Scale bars= 50 µm.

Supplementary Figure 8 Sequences of primers used in the study.

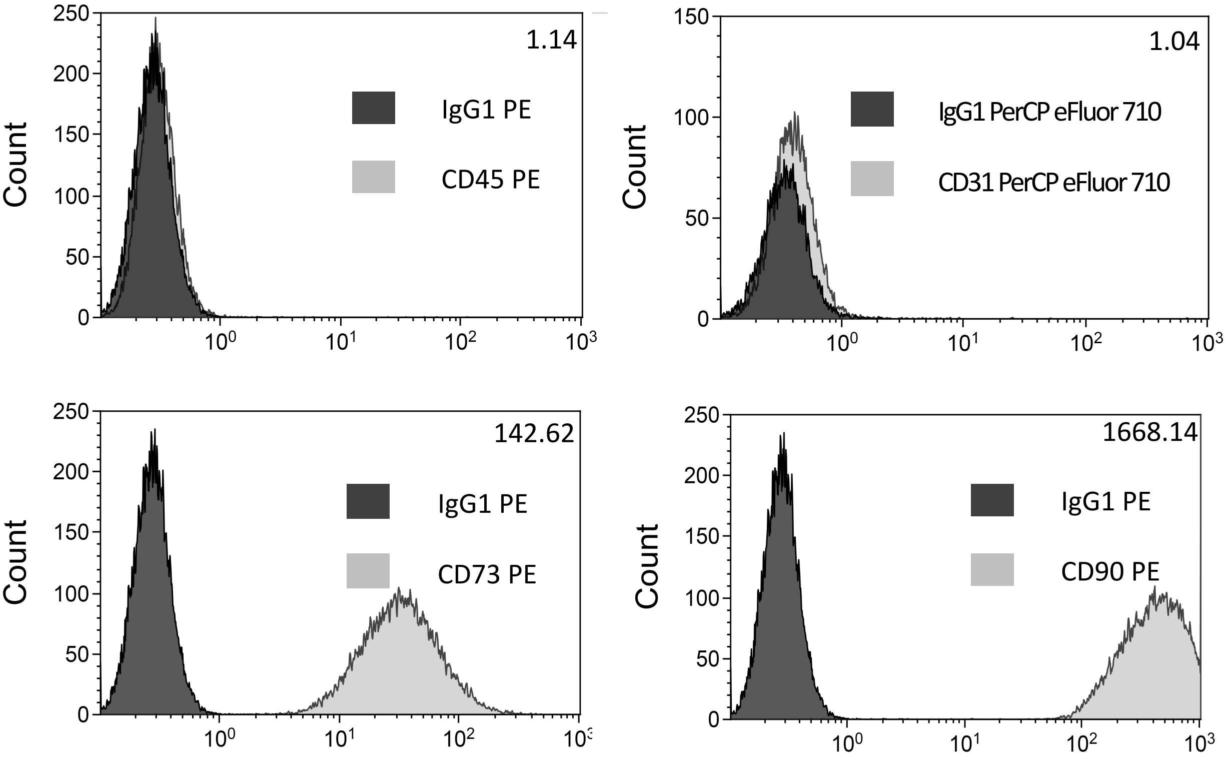

Bigot et al, Supplementary Figure 1

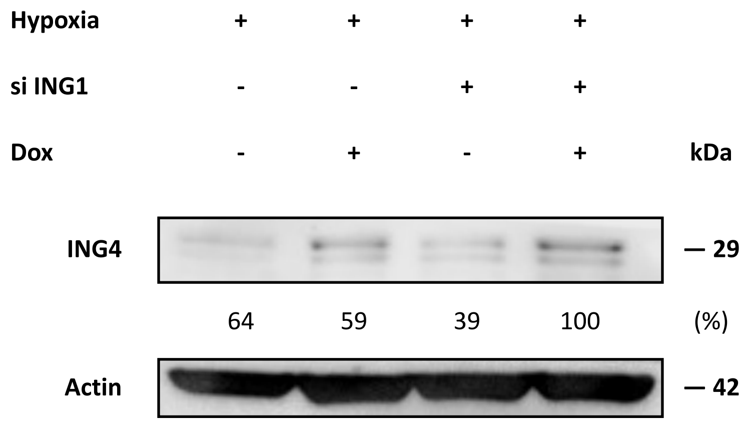

Bigot et al, Supplementary Figure 2

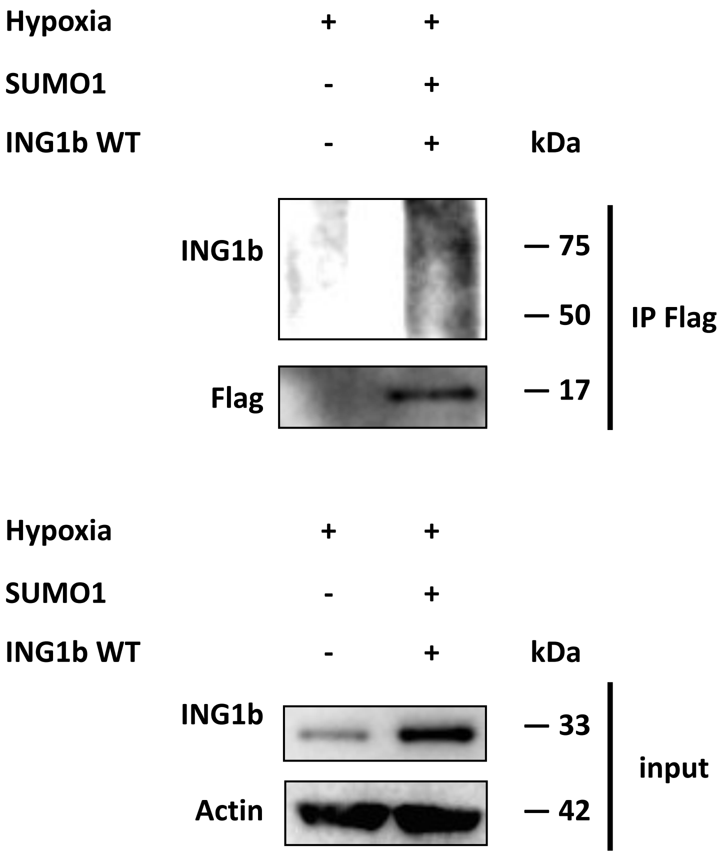

Bigot et al, Supplementary Figure 3

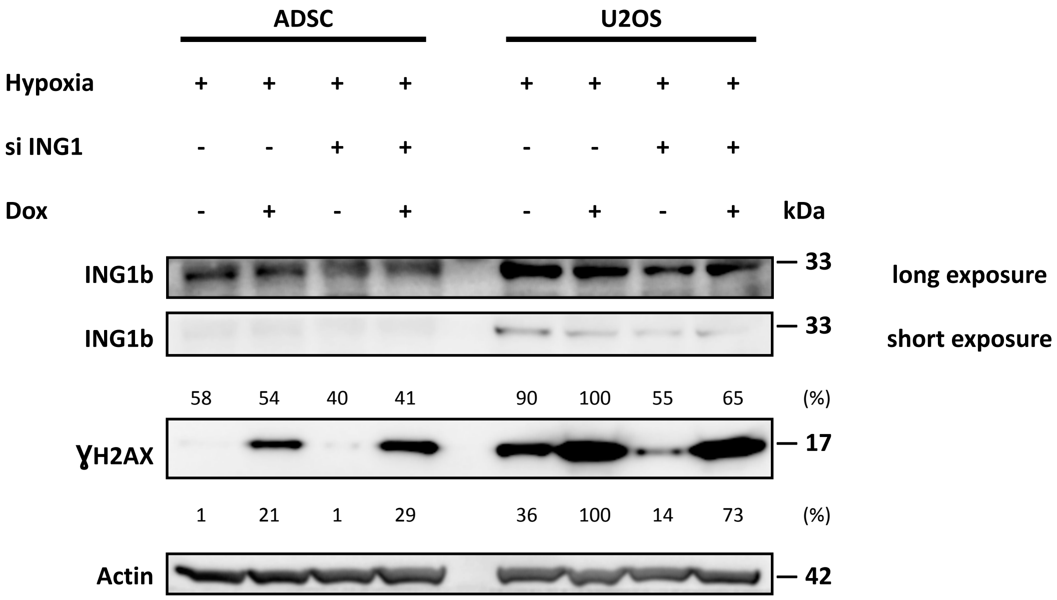

Bigot et al, Supplementary Figure 4

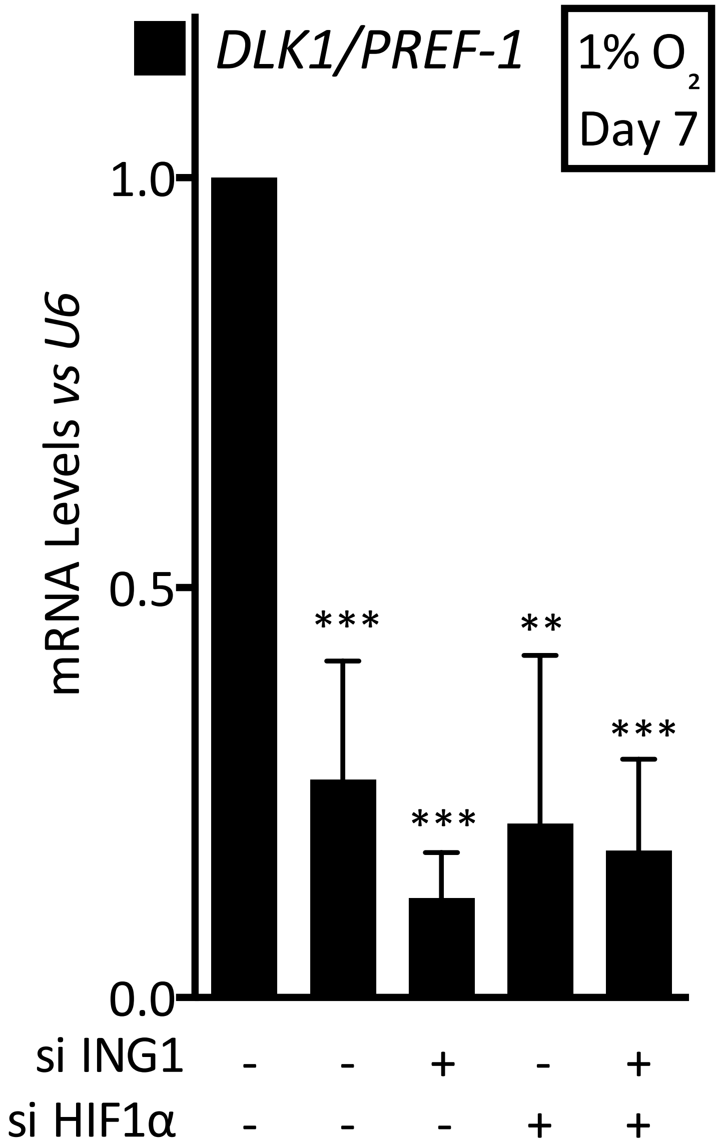

Bigot et al, Supplementary Figure 5

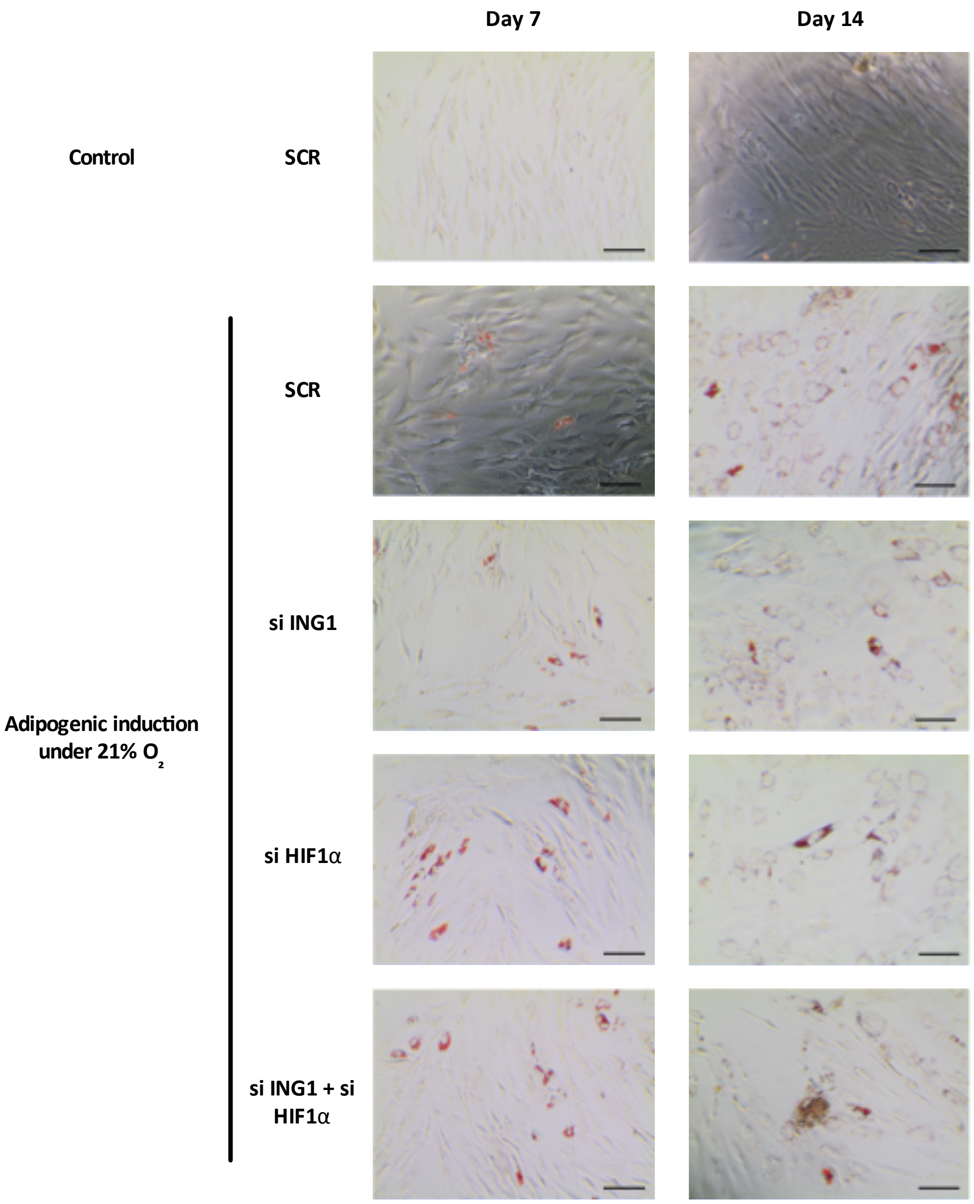

Bigot et al, Supplementary Figure 6

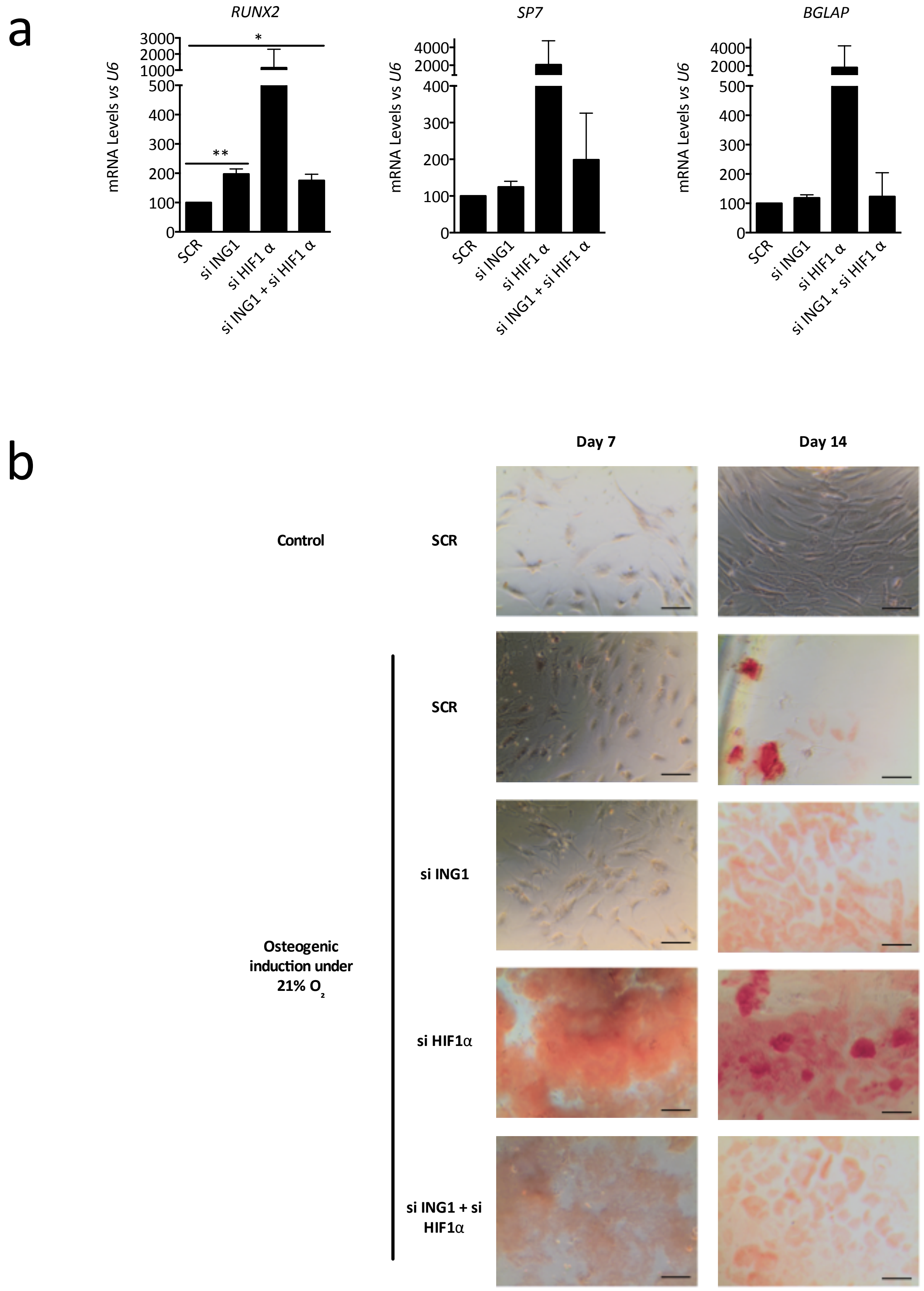

Bigot et al, Supplementary Figure 7

Primers sequences used in qRT-PCR

	Forward primer (5'3')	Reverse primer (5'3')
U6	CTCGCTTCGGCAGCACA	AACGCTTCACGAATTTGCGT
HIF1α	GAGATGTTAGCTCCCTATATCCCA	TAGGTTCTTGTATTTGAGTCTGCTG
ING1	CAACAACGAGAACCGTGAGA	GAGACCTGGTTGCACAGACA
FABP4	ATGGGGGTGTCCTGGTACAT	ACGTCCCTTGGCTTATGCTC
LPL	AGAGCCAAAAGAAGCAG	GGCAGAGTGAATGGGAT
PPARɣ2	GCAAACCCCTATTCCATGCTG	CACGGAGCTGATCCCAAAGT
RUNX2	TTCCAGACCAGCAGCACTC	CAGCGTCAACACCATCATT
BGLAP	ATGAGAGCCCTCACACTCCT	CTTGGACACAAAGGCTGCAC
SP7	TCCAGAGAGGAGAGACTCGG	GAGTTGTTGAGTCCCGCAGA
DLK1/PREF-1	CTGGACGATGGCCTCTATGAATG	ATCATCCACGCAGGTGCCTC
